# Probing the Molecular Mechanisms in Copper Amine Oxidases by Generating Heterodimers

**DOI:** 10.1002/cbic.201402653

**Published:** 2015-01-21

**Authors:** Thembaninkosi G Gaule, Mark A Smith, Arwen R Pearson, Peter F Knowles, Michael J McPherson

**Affiliations:** [a] Astbury Centre for Structural Molecular Biology, School of Molecular and Cellular Biology, Faculty of Biological Sciences, University of Leeds LS2 9JT Leeds (UK) E-mail: m.j.mcpherson@leeds.ac.uk; [b] Hamburg Centre of Ultrafast Imaging, University of Hamburg, CFEL Building 99, Luruper Chausse 149, 22761 Hamburg (Germany)

**Keywords:** *Arthrobacter globiformis*, cooperativity, copper amine oxidases, heterodimers

## Abstract

For some homodimeric copper amine oxidases (CuAO), there is suggestive evidence of differential activity at the two active sites implying potential cooperativity between the two monomers. To examine this phenomenon for the *Arthrobacter globiformis* CuAO (AGAO), we purified a heterodimeric form of the enzyme for comparison with the homodimer. The heterodimer comprises an active wild-type monomer and an inactive monomer in which an active-site tyrosine is mutated to phenylalanine (Y382F). This mutation prevents the formation of the trihydroxyphenylalanine quinone (TPQ) cofactor. A pETDuet vector and a dual fusion tag strategy was used to purify heterodimers (WT/Y382F) from homodimers. Purity was confirmed by western blot and native PAGE analyses. Spectral and kinetic studies support the view that whether there are one or two functional monomers in the dimer, the properties of each functional monomer are the same, thus indicating no communication between the active sites in this bacterial enzyme.

Copper amine oxidases (CuAOs) [E.C. 1.4.3.6] have been studied since 1929.[1] They are homodimers with subunit sizes ranging from 70 to 120 kDa.[2] Each subunit contains a single post-translationally derived protein cofactor, 2,4,5-trihydroxyphenylalanine quinone (TPQ), and a mononuclear type II cupric ion centre. The biogenesis of TPQ in CuAOs is catalysed by a single turn-over reaction from an active-site tyrosine residue in the presence of Cu^II^ and molecular oxygen.[3] CuAOs catalyse the oxidative deamination of primary amines to their corresponding aldehydes with a concomitant release of NH_3_ and H_2_O_2_ through reductive and oxidative half reactions.

Previous studies have demonstrated that CuAOs are only functional in their dimeric form; this suggests that dimerisation affords structural and/or functional integrity.[4] Further studies have provided evidence for communication between the subunits in the dimer in some CuAOs, though this evidence cannot be regarded as definitive.[5] The initial evidence came from the results of titration of the TPQ cofactor with hydrazine-based mechanistic inhibitors.[5a] These studies demonstrated that, in some CuAOs, such as pea seedling amine oxidase (PSAO) and lentil seedling amine oxidase (LSAO), two TPQs were readily titratable; this is consistent with no communication between the two active sites.[5b], [6] There are conflicting reports regarding differential reactivity between the two TPQs in bovine serum amine oxidase (BSAO).[5c–e] Morpurgo and colleagues report differential reactivity of the two TPQ's in the BSAO dimer.[5d,e] By contrast, for highly purified BSAO, Janes and Klinman demonstrated titration of up to 0.9 TPQ/subunit with corresponding enzyme activity, thereby indicating that each subunit contains active TPQ although, as the authors state, this does not necessarily mean that under steady-state conditions each subunit is catalytically competent.[5c] Choi et al. reported a titration of 0.62 TPQ/subunit with phenylhydrazine for *Arthrobacter globiformis* amine oxidase (AGAO). However, they were unable to say whether less than one TPQ per subunit is formed or if there is reduced reactivity of one of the TPQ's in the dimer as a consequence of modification of the other TPQ by the inhibitor.[5f]

In other amine oxidases such as pig plasma amine oxidase (PPAO)[5a] and *Aspergillus nidulans* amine oxidase (ANAO),[4] differential reactivity between the two TPQs has been reported, thus suggesting the possibility of communication between the active sites. In support of these observations, structural studies of *Escherichia coli* amine oxidase (ECAO) in complex with 2-hydrazinopyridine (2-HP), revealed that one of the TPQ sites reacted much more quickly than the second site, which required prolonged exposure to 2-HP to react.[7] These structural data correlated with solution studies that demonstrated that 1.5 TPQs could be titrated with molar equivalent amounts of 2-HP. However, two TPQs per dimer could be titrated following incubation with a 20-fold molar excess of inhibitor.[7a] This implied that a population of TPQ was initially inaccessible and raised the possibility of negative cooperativity between the two active sites. The most obvious, and significant, interaction between the monomers in the CuAO dimer occurs through two long β-hairpin structures: arm I and arm II. Arm I consists of residues conserved across the CuAO enzyme family and it extends from one monomer to form a network of hydrogen bonds in close proximity to the active site of the other monomer (Figure 1).[2f] Mutagenesis studies on *A. globiformis* histamine oxidase showed that altering a conserved aspartate to glutamine—D403Q (=D383 in AGAO)—increased the turnover rate of the enzyme.[8] Furthermore, characterisation of a similar residue in *Hansenula polymorpha* amine oxidase-1 (HPAO-1)—E406 (=D383 in AGAO, Figure 1)—revealed that the hydrogen-bond network has a role in both TPQ biogenesis and catalysis.[9] There is no evidence at this time for a role for these interactions in cooperativity between subunits in AGAO.

**Figure 1:**
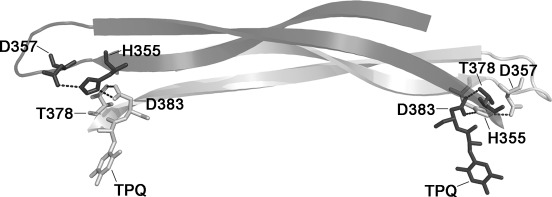
β-Hairpins (arm I) linking the active sites of AGAO monomers through a conserved hydrogen bond. β-Strands are drawn as flat arrows coloured light grey for monomer A and dark grey for monomer B. Some key residues in the active site are drawn as sticks coloured by chain. Apart from D383, the residues (T378, D357 and H355) are not referred to in the text. Hydrogen bonds are represented by dashed black lines.

Mutating the residues associated with the inter-subunit interactions within one monomer of the dimer might afford a better understanding of the relationship between dimerisation and enzyme activity in CuAO through studying heterodimeric forms of the enzyme. The most common method used to form heterodimers of enzymes with differentially mutated subunits involves dissociating two forms of homodimer, followed by random re-association of monomers giving rise to three dimer combinations: the two original homodimers plus heterodimers.[10] However, reported heterodimer yields have been low. Recent studies have demonstrated more efficient methods for obtaining higher yields of the desired heterodimer.[10] Ohgari et al. coexpressed wild-type and mutant proteins by using the pETDuet system to give 25 % wild-type dimer, 50 % heterodimer and 25 % mutant dimer yield. Castellani et al. used a dual-affinity-tag system to isolate heterodimer; this ensured low cross contamination of the heterodimer with either homodimer.[11]

In order to study the reported cooperativity in CuAOs more rigorously, we combined these methods. Initially we tested the *E. coli* enzyme, but this proved technically challenging, so we developed a coexpression and purification system for heterodimeric forms of AGAO as a model system for this proof-of-principle study. We demonstrate here the use of a coexpression vector, pETDuet-1, to isolate heterodimers comprising differentially tagged wild-type monomer and inactive Y382F monomer, in which the TPQ precursor Tyr382 is mutated to phenylalanine, are combined (Scheme 1).

**Scheme 1:**
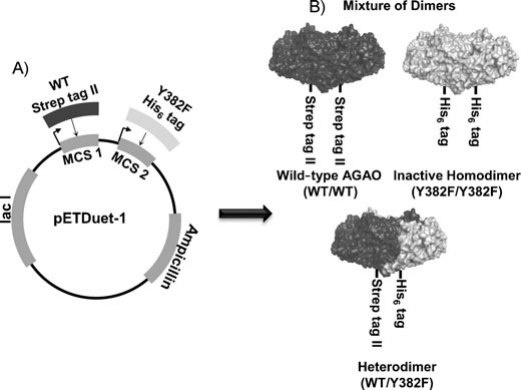
Production of AGAO heterodimers. A) C-terminal Strep-tagged WT and C-terminal His_6_-tagged Y382F genes were cloned into pET-Duet-1. B) Protein expression from pETDuet-*agao* WT/Y382F produced three dimeric forms of the enzyme.

Methods described by Ohgari et al. and Juda et al. were adapted for the construction and expression of AGAO heterodimers.[11b], [12] Briefly, a C-terminal Strep-tag II version of the wild-type *agao* coding region (provided by Prof. David Dooley, University of Rhode Island) and a C-terminal His_6_-tagged *agao* Y382F coding region were subcloned into multiple cloning regions 1 and 2, respectively, to give plasmid pETDuet-agaoWT/Y382F. Expression of protein from pETDuet-agaoWT/Y382F results in three potential dimer species (Scheme 1).

To isolate heterodimeric WT/Y382F, a two-step affinity purification procedure was employed: an initial Cu^2+^-immobilised affinity chromatography step to remove WT dimers and then a StrepTactin affinity chromatography step to remove Y382F homodimers. Wild-type AGAO (WT/WT) and the inactive mutant (Y382F/Y382F) were purified separately as experimental controls. The purification of heterodimeric or homodimeric populations together with experimentally mixed homodimers allowed for direct and rapid confirmation by western blot analysis of the subunit composition. Immunoblotting with an anti-Strep tag II antibody detected both WT/WT and WT/Y382F AGAO dimers, whereas Y382F/Y382F and WT/Y382F AGAO dimers were detected by an anti-polyhistidine antibody. Immunoblotting for each tag (Strep tag II and His_6_ tag) independently confirmed the presence and purity of heterodimeric WT/Y382F AGAO (Figure 2).

**Figure 2:**
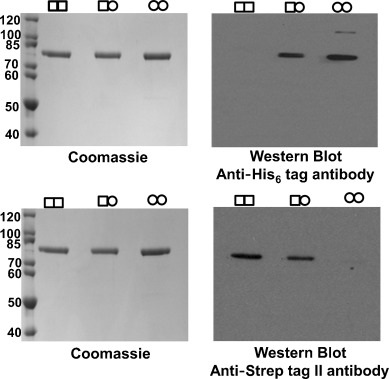
SDS-PAGE (Coomassie Brilliant Blue-stained gels) and western blot analysis of the subunit combinations: □: WT, ○: Y382F.

Theoretical calculations of isoelectric point (pI, ExPASy; http://web.expasy.org/compute_pi/) indicated that each dimer would have a different pI indicative of changes to the overall charge due to the fusion tags (WT/WT pI=5.07, WT/Y382F pI=5.15 and Y382F/Y382F pI=5.23).[13] Furthermore, under alkaline conditions the Strep tag II (WSHPQEK) is protonated whilst the His_6_ tag (HHHHHH) is deprotonated. Subsequent analysis by native PAGE at pH 8.8 demonstrated differential migration patterns for these three protein dimers, thereby confirming the predicted differences in overall protein charge and migration characteristics of the three AGAO dimers and confirming purification of the heterodimer (Figure 3). Following prolonged incubation of heterodimer protein samples with subsequent native PAGE established that there was no random dissociation and reassociation of the dimers as only a single band was observed for WT/Y382F AGAO with no evidence of the appearance of homodimers. As the native PAGE and immunoblot data demonstrated successful isolation of the heterodimeric WT/Y382F, spectral and steady-state kinetic studies were undertaken. The presence of protein-derived cofactor, TPQ, provides a clear spectral signature with *λ*_max_=480 nm.[3b] UV–visible spectra of purified WT/WT, WT/Y382F and Y382F/Y382F AGAO dimers revealed an absorption peak at 480 nm for WT/WT and WT/Y382F samples but not for the Y382F/Y382F AGAO variant, which cannot form TPQ. The molar extinction coefficient for WT/Y382F (1752 m^−1^ cm^−1^) is half that of WT/WT (3437 m^−1^ cm^−1^); this indicates a 50 % lower TPQ content. The absence of shifts in *λ*_max_ in WT/Y382F compared with WT/WT indicates that there is no apparent change in the electronic structure of the TPQ in the heterodimer (Figure 4 A).

**Figure 3:**
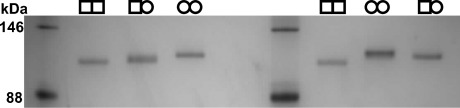
Native PAGE gel displaying the difference in migration of WT/WT (□□) AGAO, WT/Y382F (□○) AGAO and Y382F/Y382F (○○) AGAO.

**Figure 4:**
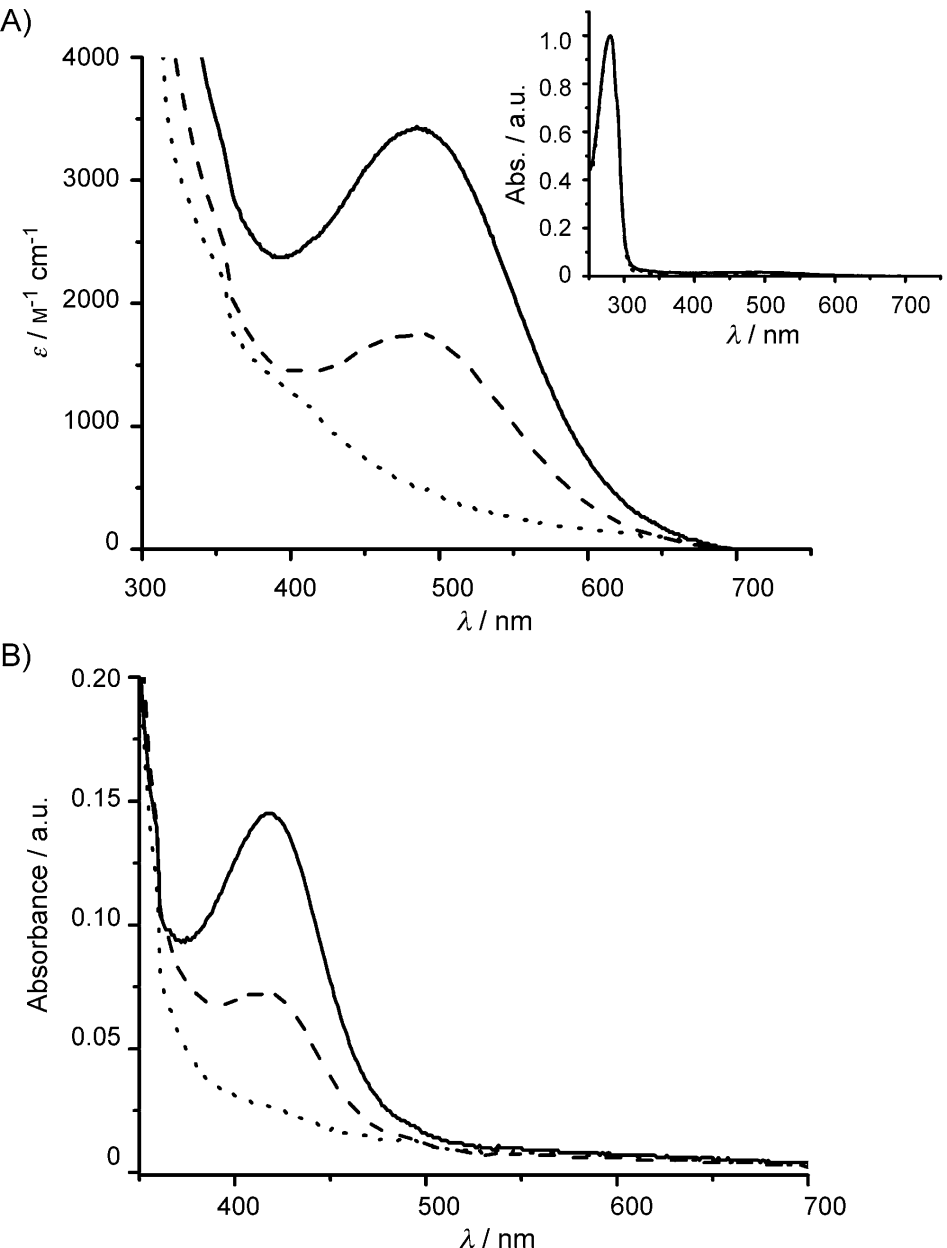
A) UV/Vis spectra of WT/WT (—), WT/Y382F (– – –) and Y382F/Y382F AGAO (⋅⋅⋅⋅; all 64 μm) in HEPES buffer (50 mm, pH 7.0). B) Adduct I UV/Vis spectra of WT/WT, WT/Y382F and Y382F/Y382F AGAO (all 26 μm) supplemented with 2-HP (0.52 mm) in potassium phosphate (100 mm, pH 7.0).

To support the UV–visible data, the WT/WT, WT/Y382F and Y382/Y382F dimers were incubated with a 20-fold molar excess of 2-HP to react with the TPQ in each protein. 2-HP is a mechanism-based inhibitor of CuAOs (Scheme 2)[5a] that gives the yellow coloured complex, adduct **I**.[14] A change in solution colour from pink (unreacted TPQ at 480 nm) to yellow (adduct **I** complex at 420 nm) was only observed with WT/WT and WT/Y382F AGAOs, but not with Y382F/Y382F, as expected (Figure 4 B).

**Scheme 2:**
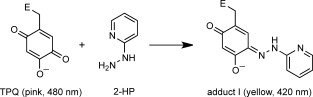
The irreversible reaction of TPQ with 2-HP.

Spectrophotometric quantification of adduct **I** in equal quantities of protein revealed that heterodimeric WT/Y382F AGAO (0.072 a.u) formed only half the quantity of adduct **I** than the WT/WT AGAO (0.145 a.u) homodimer did. Post-translational modification of Y382 to TPQ in the WT subunit of the WT/Y382F AGAO heterodimer indicates that the Y382F mutation of in one monomer has no effect upon TPQ formation in the other.

To quantify the content of TPQ in active WT/WT, inactive Y382F/Y382F and heterodimeric WT/Y382F AGAOs, the enzymes were titrated with 2-HP. This indicated 1.5 TPQs per dimer of the WT/WT enzyme and is consistent with the 1.4 TPQs per dimer reported previously.[12] Many questions have been raised about the failure to detected one TPQ per monomer in many CuAOs.

Does this population exist as TPQ or an unreactive precursor? Does the conformation of the active site prevent a population reacting or does inter-subunit communication lead to a conformation change in the neighbouring active site? In heterodimeric WT/Y382F AGAO, 0.8 TPQs were titrated per dimer and, as expected from the results with excess 2-HP, no TPQ was detected upon titration of the inactive homodimer Y382F/Y382F (Figure 5). The data show that heterodimeric WT/Y382F has approximately half the quantity of reactive TPQs of the WT/WT AGAO. Ruggiero and Dooley observed 70–80 % efficiency in TPQ biogenesis in AGAO, as the amount of oxygen consumed correlated to the amount of TPQ formed, typically 0.65–0.85 mol of TPQ per mol of subunit.[15] Taking this into account, it seems most likely that about 20 % of the precursor tyrosine fails to undergo full processing to form the cofactor TPQ, resulting in the detection of less than one TPQ in the heterodimer WT/Y382F.

**Figure 5:**
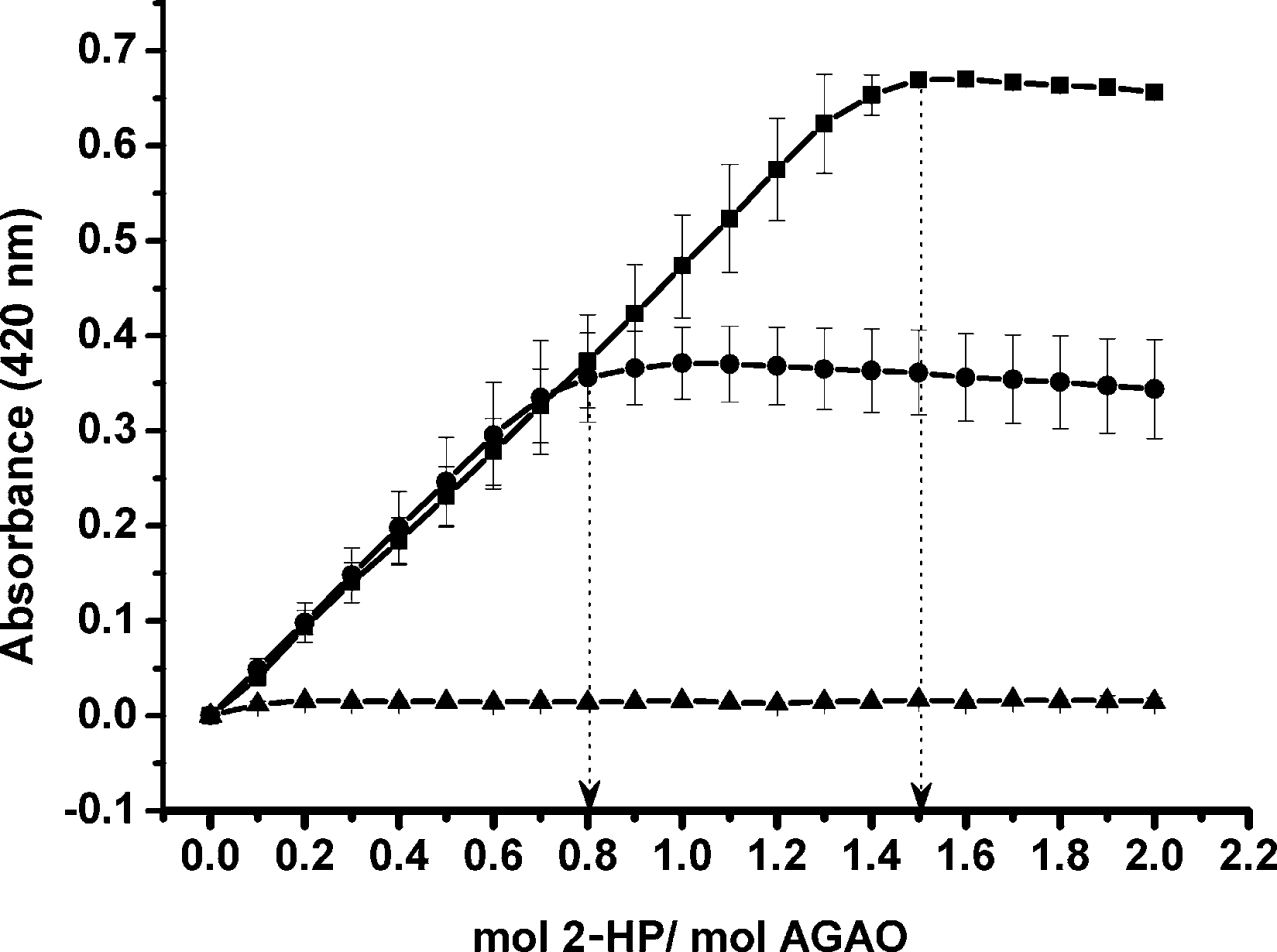
TPQ titrations of WT/WT (▪), WT/Y382F (•) and Y382F/Y382F (▴) AGAO with 2-hydrazinopyridine. The absorbance decrease observed at high molar ratios of 2-HP to AGAO is due to the slow conversion of the hydrazone form of adduct I to adduct II. Spectra are the average of three measurements, and absorbance values were corrected for progressive dilution caused by addition of 2-HP.

The catalytic activity of AGAO was assessed in a peroxidase-coupled assay, as described in Chiu et al.,[16] with β-phenylethylamine (β-PEA) as the substrate. The *K*_M_ values for β-PEA of WT/WT and WT/Y382F AGAOs are similar (1.3±0.1 vs. 1.6±0.2 μm, respectively). Oxidation of β-PEA also displays substrate inhibition in 100 mm HEPES (pH 7.0),[17] and therefore a similar trend was observed upon comparison of the *K*_i_ values of the WT/WT and WT/Y382F AGAOs (198.2±35.7 vs. 208.2±26.9 μm, respectively; Table 1).[17] This suggests that inactivating one monomer has no effect on either substrate binding or substrate inhibition in the active monomer. The turnover rate (*k*_cat_) of WT/Y382F AGAO per dimer is 65.5±1.9 s^−1^, which is approximately half that of WT/WT AGAO (129.5±5.1 s^−1^). Although heterodimeric WT/Y382F AGAO exhibits half the total catalytic efficiency of the WT/WT AGAO homodimer, due to one inactive subunit (4×10^7^ vs. 9.96×10^7^ m^−1^ s^−1^, respectively), the catalytic efficiencies per wild-type monomer in heterodimer or homodimer AGAO are similar (Table 1).

**Table 1:** Kinetic parameters of AGAO dimers for β-phenylethylamine[a]

Dimer	*K*_M_ [μm]	*k*_cat_ [s^−1^]	*K*_i_ [μm]	*k*_cat_/*K*_M_ [m^−1^ s^−1^] (dimer)
WT/WT	1.3 (±0.1)	129.5 (±5.1)	198.2 (±35.7)	9.96×10^7^
				(±8.6×10^6^)
WT/Y382F	1.6 (±0.2)	65.5 (±1.9)	208.2 (±6.9)	4×10^7^
				(±5.3×10^6^)

[a] Assays conducted under air-saturating conditions at 25 °C in HEPES (50 mm, pH 7.0). No turnover was detected for the inactive Y382F/Y382F variant.

The steady-state kinetic data are in agreement with the TPQ titration data thus indicating that inactivating one active site in AGAO has no effect on the reactivity of a second functional active site. The TPQ titration data imply that inefficiency in TPQ biogenesis results in the formation of less than two TPQs in AGAO. However, we cannot exclude the possibility that a population of TPQ is inaccessible to 2-HP. That inactivating one subunit has no significant effect on the activity of the active subunit suggests that, in AGAO, the subunits act independently of each other. This suggests that replacing the TPQ in one subunit with phenylalanine does not appear to change the hydrogen-bond network involved in interactions with the β-hairpin, and, consequently, the activity in the other subunit is unaffected. The independent catalytic activity of each subunit suggests that dimerisation is likely to confer structural stability on AGAO. However, continued study of other heterodimers variants will allow for more detailed investigations into the function of dimerisation in AGAO and in other CuAOs.

In conclusion, we report purification of the first heterodimeric form of a CuAO. The data suggest that the subunits are catalytically independent in AGAO, thus there is no subunit cooperativity. Heterodimeric forms of CuAOs will allow the study of differentially mutated subunit combinations within the dimer and will further facilitate the dissection of structural features associated with enzyme function.

## Experimental Section

**Cloning:** The C-terminal Strep-tagged wild-type AGAO gene was PCR amplified from pAGO2 and subcloned into pET28c. Prior to constructing pET28c_Y382F, the pET28c_WTAGAO construct was altered to remove an internal NcoI site by a single base mutation at base pair 1377 (C→G); this maintained the same codon using the following primers: reverse primer: 5′-GTGGT CCGGC AAAC**G** ATGGG CCCGG GC-3′, Forward primer: 5′-GCCCG GGCCC AT**C**GT TTGCC GGACC AC-3′. pET28c_Y382F was constructed by using site-directed mutagenesis with the following primers in which the mutated nucleotide is shown in bold, underlined; forward primer: 5′-CACCA CTATC GGCAA CT**T**CG ACTAC GGCTT CTACT GG-3′ and reverse primer: 5′-GTGGT GATAG CCGTT G**A**AGC TGATG CCGAA GATGA CC-3′.

The Y382F and WT AGAO coding genes were subcloned into the coexpression vector, pETDuet-1. The Strep-tagged WTAGAO coding region was amplified by PCR with the following primers: 5′-CATG**C CATGG** GCACG CCCTC CACTA TCCAA ACAGC-3′ and 5′-CCC**AA GCTT**G GGTCA TTTCT CAAAC TGCGG-3′ which introduced unique restriction sites, NcoI and HindIII (bold, underlined) respectively. The amplified fragment was subcloned into multiple cloning site-1 (MCS1) of pETDuet-1.

The same procedure was used for the Y382F gene. The Y382F coding gene was amplified by PCR using the following primers: forward primer: 5′-GGAAT TC**CAT ATG**AC GCCCT CCACT ATCCA AACAG C-3′ and reverse primer: 5′-CCG**CT CGAG**C GGTCA GTGAT GATGA TGATG ATGCC GTGGC AGTGG GAGCC-3′ which introduced unique restriction sites NdeI And XhoI (bold, underlined), respectively. The reverse primer also introduced a His-tag site that replaced the Strep-tag site. The fragment was subcloned into multiple cloning site-2 (MSC2) of coexpression vector pETDuet-1. The Strep-tagged WT AGAO and His_6_-tagged Y382F coding regions were subcloned both separately and together into pETDuet-1 to form pETDuet-WT, pETDuet-Y382F and pETDuet-WT/Y382F. Each construct was confirmed by diagnostic restriction enzyme digests followed by DNA sequencing.

**Protein expression and purification:** The AGAO enzymes were expressed as described in Juda et al.[12] All the purification steps were carried out at 4 °C. Post-lysis, CuSO_4_ (50 μm) was added to the lysates, and they were incubated at 30 °C for 1 h with shaking at 100 rpm. This step was performed to ensure the full processing of tyrosine to TPQ. Excess CuSO_4_ was removed by dialysing the lysate against binding buffer phosphate-buffered saline (PBS, 140 mm NaCl, 2.68 mm KCl, 10 mm Na_2-HP_O_4_, 2 mm KH_2_PO_4_, pH 7.4) or sodium phosphate (20 mm containing 300 mm NaCl, pH 7.4). Wild-type AGAO (WT/WT) was purified by using Strep-Tactin chromatography.[12] The column was prepared according to the manufacturer's protocol (Strep-Tactin Sepharose 50 % suspension, IBA, Göttingen, Germany). The lysate was syringe filtered prior to loading the Strep-Tactin column. The protein-bound column was washed with binding buffer to remove unbound and non-specific binding proteins. The enzyme was eluted with PBS supplemented with [D]desthiobiotin (5 mm). Homogeneous fractions were pooled and dialysed against HEPES (50 mm, pH 7).

The inactive mutant (Y382F/Y382F) was purified by using Cu^2+^-immobilised metal affinity chromatography. The dialysed lysate was filtered through a 0.2 μm syringe filter to remove any remaining cell debris. Prior to sample loading, the column was charged with NiSO_4_ (0.1 m) and equilibrated with sodium phosphate (20 mm containing 300 mm NaCl, pH 7.4). After the sample had been loaded, the column was washed with sodium phosphate (20 mm containing 300 mm NaCl, 40 mm imidazole, pH 7.4). Bound protein was eluted with sodium phosphate (20 mm containing 300 mm NaCl, 250 mm imidazole, pH 7.4).Homogenous fractions were then pooled and dialysed against HEPES (50 mm, pH 7).

The heterodimer (WT/Y382F) was isolated according to a two-step affinity purification procedure that involved both Cu^2+^-immobilised affinity chromatography and Strep-Tactin affinity chromatography. The column was charged with CuSO_4_ (0.1 m), washed to remove unbound Cu^2+^ and equilibrated with sodium phosphate (20 mm), NaCl (300 mm) pH 7.4. The protein-bound column was washed with sodium phosphate (20 mm containing 500 mm NaCl, 40 mm imidazole, pH 7.4). Elution was carried out using sodium phosphate (20 mm containing 500 mm NaCl, 50 mm EDTA, pH 7.4). One fraction was collected, analysed by SDS-PAGE and dialysed against PBS in preparation for Strep-Tactin chromatography. The purification procedure was similar to that used for WT/WT. The purity and homogeneity of the eluted protein was assessed by SDS-PAGE, and the protein was dialysed overnight against HEPES (50 mm, pH 7).

**Western blot:** After being separated on a SDS-PAGE (15 %) gel, the proteins were transferred to two identical polyvinylidene fluoride (PVDF) membranes (Immobilon, Millipore) by using an XCell II module apparatus (Invitrogen). Following transfer, the membranes were blocked in dried skimmed milk (Marvel, 5 %, *w*/*v*) in Tris-buffered saline (150 mm NaCl, 50 mm Tris**⋅**HCl, pH 7.5) containing Tween-20 (TBS-T, 0.1 %) and bovine serum albumin (BSA, 5 %, *w*/*v*) in TBS-T for 1 h. One membrane was then incubated overnight at 4 °C in TBS-T with dried skimmed milk (5 %, *w*/*v*) and supplemented with the monoclonal anti-polyhistidine peroxidise conjugate antibody (Sigma); the other membrane was incubated in BSA (5 %, *w*/*v*) and TBS-T containing the primary anti-Strep tag II antibody (Novagen). After incubation, the unbound antibody was washed off in TBS-T. The second membrane was then incubated for 1 h in BSA (5 %, *w*/*v*) and TBS-T supplemented with a horseradish peroxidase-conjugated secondary antibody (Sigma) for 1 h. The unbound secondary antibody was washed off with TBS-T. The proteins were detected by using chemiluminescence.

**Native PAGE:** The traditional Tris–glycine system was used; the proteins were separated by using a native polyacrylamide gel (12 %). Electrophoresis was carried out at 4 °C and 200 V for 1–2 h, and the proteins were detected by staining with Coomassie Brilliant Blue R.

**Kinetic assays:** AGAO activity was assessed by use of the peroxidase-coupled assay described by Chiu et al.[16] Briefly, enzyme activity was assayed at 30 °C in HEPES buffer (100 mm, pH 7) with various concentrations of β-phenylethylamine (0.5–160 μm). The reactions were followed by monitoring H_2_O_2_ production, which oxidises 2,2′-azinobis(3-ethylbenzthiazoline-6-sulfonic acid) (ABTS; 2 mm; *ε*_414_=24 600 m^−1^ cm^−1^) using horseradish peroxidase (HRP; 10 units mL^−1^). The data were fitted to Equation 1 by using Origin Pro 7.5 (Microcal, MA, USA) [Eq. (1)].[17]

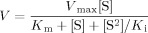
(1)

**UV–visible spectroscopic studies and TPQ titrations:** UV–visible spectra were obtained on a Shimadzu UV2401 PC spectrophotometer equipped with a temperature-controlled cell holder. The proteins were treated with a 20-fold molar excess of 2-HP in HEPES (100 mm, pH 7). Spectral changes associated with this addition of 2-HP to WT/WT, Y382F/Y382F and WT/Y382F AGAO were monitored over time, typically 5–10 min, until there were no further changes. TPQ titrations were carried out as previously described. 2-HP was prepared at a molar concentration ten times that of the dimer. TPQ was titrated stepwise by adding 2-HP (0.1 equiv). Changes in absorbance were accounted for by correcting for the dilution (1 %) at each addition of 2-HP. Following addition of 2-HP, the reactions were allowed to proceed until no detectable change in absorbance was observed.

## Abbreviations

AGAO: *Arthrobacter globiformis* amine oxidase
ANAO: *Aspergillus nidulans* amine oxidase
BSAO: bovine serum amine oxidase
CuAO: copper amine oxidase
ECAO: *Escherichia coli* amine oxidase
2-HP: 2-hydrazinopyridine
HPAO: *Hansenula polymorpha* amine oxidase-1
LSAO: lentil seedling amine oxidase
β-PEA: β-phenylethylamine
PPAO: pig plasma amine oxidase
PSAO: pea seedling amine oxidase
TPQ: 2,4,5-trihydroxyphenylalanine quinone
